# Supporting the clinical use of the ICF in Japan – development of the Japanese version of the simple, intuitive descriptions for the ICF Generic-30 set, its operationalization through a rating reference guide, and interrater reliability study

**DOI:** 10.1186/s12913-020-4911-6

**Published:** 2020-01-30

**Authors:** Masahiko Mukaino, Birgit Prodinger, Shin Yamada, Yuki Senju, Shin-Ichi Izumi, Shigeru Sonoda, Melissa Selb, Eiichi Saitoh, Gerold Stucki

**Affiliations:** 1 Department of Rehabilitation Medicine I, Fujita Health University School of Medicine, Toyoake, Aichi Japan; 2 Faculty of Applied Health and Social Sciences, Technical University of Applied Sciences, Rosenheim, Germany; 3 Swiss Paraplegic Research, Nottwil, Switzerland; 4 ICF Research Branch, a cooperation partner within the WHO Collaborating Centre for the Family of International Classifications in Germany (at DIMDI), Nottwil, Switzerland; 5 Department of Rehabilitation Medicine, Kyorin University School of Medicine, Tokyo, Japan; 6 Department of Rehabilitation, Ise Municipal General Hospital, Ise, Mie Japan; 7 Department of Physical Medicine and Rehabilitation, Tohoku University Graduate School of Medicine, Sendai, Miyagi Japan; 8 Department of Rehabilitation Medicine II, School of Medicine, Fujita Health University School of Medicine, Tsu, Mie Japan; 9 Department of Health Sciences and Medicine, University of Luzern, Luzern, Switzerland

**Keywords:** ICF, ICF rehabilitation set, Simple, intuitive descriptions, Interrater reliability

## Abstract

**Background:**

The World Health Organization developed the International Classification of Functioning, Disability, and Health (ICF) in 2001 and has been in the process of implementing it in clinics since then. Current international efforts to implement ICF in rehabilitation clinics include the implementation of ICF Core Sets and the development of simple, intuitive descriptions for the ICF Generic-30 Set (also called Rehabilitation Set). The present study was designed to operationalize these ICF tools for clinical practice in Japan. This work included 1) the development of the Japanese version of the simple, intuitive descriptions for the ICF Generic-30 Set, 2) the development of a rating reference guide for Activity and Participation categories, and 3) the examination of the interrater reliability of rating Activity and Participation categories.

**Methods:**

The Japanese version of the simple, intuitive descriptions for the ICF Generic-30 Set was developed following the process employed to develop the Chinese and Italian versions.

For further operationalization of this ICF Set in practice, a rating reference guide was developed. The development of the rating reference guide involved the following steps: 1) a trial of rating patients by several raters, 2) cognitive interviewing of the raters to analyse the thinking process involved in rating, 3) drafting of the rating reference guide, and 4) review by ICF specialists to confirm consistency with the original ICF concepts.

After the rating reference guide was developed, interrater reliability of the rating with the reference guide was determined. Interrater reliability was examined using weighted kappa statistics with linear weight.

**Results:**

Through the pre-defined process, the Japanese version of the simple, intuitive descriptions for 30 categories of the ICF Generic-30 Set and the rating reference guides for 21 Activity and Participation categories were successfully developed. The weighted kappa statistics ranged from 0.61 to 0.85, showing substantial to excellent agreement of the ratings between raters.

**Conclusions:**

The present study demonstrates that ICF categories can be translated into clinical practice. Collaboration between clinicians and researchers would further enhance the implementation of the ICF in Japan.

## Background

Rehabilitation is considered the key health strategy of the twenty-first century [1]. The aim of rehabilitation is to optimize functioning. Functioning, as described by the World Health Organization (WHO) in the International Classification of Functioning, Disability, and Health (ICF), refers to the interaction of body functions and structures, activities and participation with contextual factors, including environmental and personal factors [2]. Comprehensive and universally accepted, the ICF serves as the international standard for describing functioning.

Though the ICF is accepted by all WHO-member states, its application in clinical practice is still limited [3]. One reason for this is the lack of clinical tools that use language familiar to health professionals and at the same time consistent with the original concept of the ICF itself. Thus, to facilitate ICF implementation in clinical practice, health practitioner-friendly ICF-based tools have to be developed.

The first challenge is to determine *what to assess*, that is, which ICF categories to include in a data collection tool for clinical use. For this process, ICF Core Sets have been developed based on a multi-stage international consensus-process [4]. In addition to health-condition or setting specific ICF Core Sets, two Generic ICF Sets have been created [5, 6]. The ICF Rehabilitation Set (also called ICF Generic-30 Set; will be referred to ICF Generic-30 Set from now on) includes 30 ICF categories – 9 from the component body functions and structures and 21 from the activities and participation component – and is recommended for use in rehabilitation practice. Seven of the ICF Generic-30 Set categories comprise the ICF Generic-7 Set. The content validity of these selected categories has been confirmed in a previous study [7]. Though the ICF Sets assist in the process of defining what to assess, ICF categories alone are not clinical tool items. Conceptually broad ICF categories, such as d240 Handling stress and other psychological demands or d920 Recreation and leisure, can be difficult to rate if they are not further specified. Furthermore, the description of many ICF categories is not intuitive, thus precluding healthcare providers from using them on a daily basis in clinics. To address this particular issue, there have been international efforts to develop ‘simple, intuitive descriptions’ of ICF categories of the ICF Generic-30 Set [8, 9]. These simplifications are aimed at highlighting the core concepts of the original ICF category definitions in a user-friendly language to facilitate the use of ICF as a clinical tool. Once it is determined *what to assess*, the next challenge is *how to assess* single ICF categories. The ICF provides a coding scheme from 0 No problem to 4 Complete problem based on a percentage distribution, e.g. 5 to 24% limitation or restriction indicates mild problem, 25 to 49% indicates moderate problem, etc. Numerous studies have shown low interrater reliability for clinicians using the ICF qualifiers in this manner [10, 11]. Another solution is to use the qualifier structure as a rating scale from 0 to 4 without a defined percentage distribution. Psychometric evaluation of such rating scales confirmed that it works as intended, thus, supporting in principle its clinical use [12]. Although a straightforward solution is to develop detailed rating guidelines that are easier for clinicians to use, such efforts risk developing inconsistencies with the original coding guideline of the ICF. The fundamental challenge is that the ratings should be reliable between and across raters and consistent with the original principles of ICF coding.

The application of the ICF as a clinical data collection tool is important given the role of the ICF as an international standard in rehabilitation, yet is challenging due to the complexity of the ICF. As some ICF categories are multidimensional, identifying which dimension to rate may be difficult. Thus, developing a clinician-friendly rating guide would have to address such complexity in rating. Development of an ICF-based clinical data collection tool can be informed by previous work in China toward developing a tool based on the ICF Generic-7 Set for the routine use in clinical practice [13, 14]. Expanding such efforts to the ICF Generic-30 and aligning it with the routine work of rehabilitation professionals in different countries or regions would promote the implementation of ICF-based clinical data collection tools in rehabilitation practice worldwide, whereby also maintaining consistency with the original ICF. For this purpose, a systematic process for checking the consistency with the original ICF concepts and coding recommendations is needed.

The present study was designed to develop a user-friendly ICF-based clinical data collection tool for use in Japan. First, a Japanese version of simple, intuitive ICF descriptions of the ICF Generic-30 was developed in a manner that is consistent with previous studies [8, 9]. Second, we developed a simplified rating reference guide to support clinicians in rating patient functioning. Third, the interrater reliability of ratings using the simple, intuitive descriptions and the rating reference guide was investigated.

## Methods

### Development of simple, intuitive descriptions

To develop simple, intuitive descriptions based on the original ICF descriptions of the 30 categories contained in the ICF Generic-30, a consensus conference with multidisciplinary rehabilitation experts was conducted.

#### Participants

The consensus process involved 3 groups, each with 7 experts. Experts who were recruited to participate represented various clinical areas of expertise. For each group, one expert was nominated as the moderator, and an assistant from the project team was assigned to each group. The assistant was responsible for taking notes throughout the process. While the moderator had the right to vote, the assistant did not. The participants remained in their respective group throughout the entire consensus process. Two officers from the Ministry of Health, Labour and Welfare participated as observers. The spoken language at the conference was Japanese.

#### Consensus conference

**Fig. 1:**
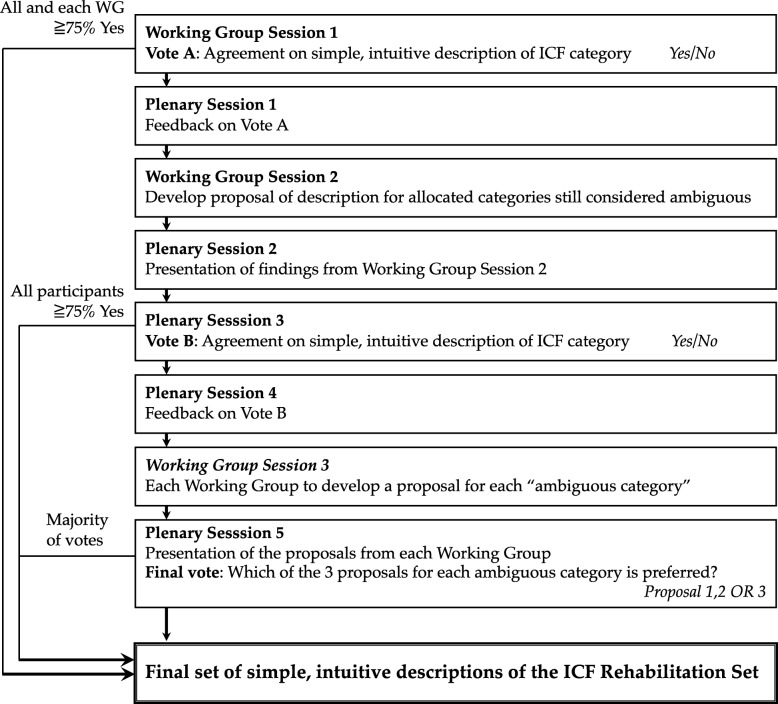
The steps of the consensus conference for developing the simple, intuitive descriptions

The conference contained three parts as illustrated in Fig. 1. Each part contained a group discussion followed by a vote. First, they were divided into three working groups (WGs). Each group received an initial proposal of simple, intuitive descriptions of the respective ICF categories. A team of clinicians developed this Japanese-language version of the simple, intuitive descriptions based on the results of the consensus conferences conducted in China and Italy [8, 9]. The participants were asked to review and discuss the initial draft proposals. Afterwards, every participant voted whether each description was simple and intuitive enough for use in routine clinical practice, while also reflecting the concept behind the original description of the corresponding ICF category, or otherwise needs revision. At the first vote, a consensus was achieved if the description achieved 75% or more agreement in each WG. After the presentations of the results and discussions in the plenary session, each category that did not reach consensus in the first vote was considered “ambiguous”. Ambiguous categories were subsequently distributed across the three different WGs, and each group was asked to propose a new description for each allocated category. In the subsequent plenary session, every proposal from the second working group session was discussed and voted upon. As in the first vote, consensus on a description was achieved when at least 75% of all participants agreed that the new description was simple and intuitive. In the third and last step of the consensus conference, each WG was asked to develop a new proposal for each of the ICF categories that continue to be ambiguous after the second vote. In the third and final plenary session, each participant was asked to vote for which of the three descriptions they preferred. The proposal with the majority vote was considered the final, simple, intuitive description for the given category. The discussion notes from the consensus conference were subsequently analysed using qualitative content analysis to enhance transparency in the decision-making process toward coming up with a given simple, intuitive description of a given ICF category. Using a modified form of the dual panel methodology [15], a multidisciplinary expert panel developed English translations from the Japanese version of the final descriptions, as previously shown [9]. The expert panel was made up of four participants from the consensus conference, who were fluent in English and Japanese, and there were three translation phases. During Phase 1, each of the four members proposed an English-language version of the descriptions. In Phase 2, the expert panel reviewed all four proposals to find a consensus on the final description. In Phase 3, the final versions of descriptions were reviewed and refined by a native English speaker.

### Development of a rating reference guide

Our next step was to create a rating reference guide that can help clinicians to use the simplified descriptions developed at the conference. A multistage development process—which included a rating trial, cognitive interviews, and an expert review—was developed to generate a rating reference guide.

#### Participants

Three rehabilitation experts from the same hospital participated in the first rating trial, the cognitive interviews, and a subsequent group discussion to develop a preliminary draft of the rating reference guide. The interviewer in the cognitive interviews also participated in the group discussion. Eight ICF experts participated in the review process to finalize the rating reference guide.

#### The consensus process for developing the rating reference guide

**Fig. 2:**
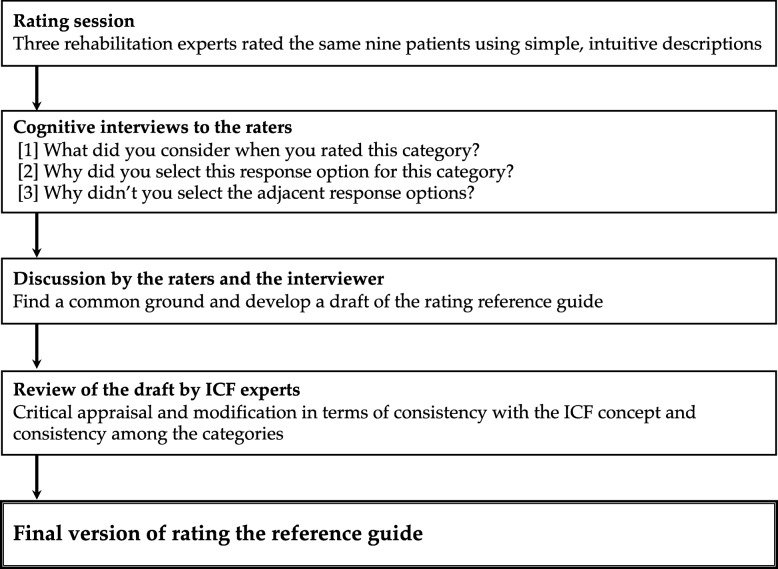
The steps of the development process of the rating reference guide

In this first study, the guide for rating categories of the activities and participation component for which simple, intuitive descriptions were agreed upon in the previous step, was developed. The development of the guide for the body function component was done in a separate project. This guide was based on cognitive interviews with rehabilitation experts in order to understand their thinking process when rating a patient (Fig. 2).

First, a multidisciplinary group of rehabilitation experts of the same hospital independently evaluated the same patients in the hospital by using the simple, intuitive descriptions. These raters were asked to select a score between zero and four according to the extent of the problems that the patients were experiencing in the given categories. Afterwards a researcher conducted a cognitive interview with each expert using verbal probing techniques [16]. The interview was composed of the following questions: [1] What did you consider when you rated this category? [2] Why did you select this response option for this category, e.g. why did you choose a rating of 2 for the category d450 Walking? [3] Why didn’t you select the adjacent response options, e.g. why didn’t you select a rating of 1 or 3 instead of a rating of 2? The raters were then asked to meet as a group to discuss each the compiled interview results and develop a simple rating reference guide accordingly. The researcher who conducted the individual interviews moderated the discussion and reference guide development process. The raters were asked to develop the reference guide for a rating scale of 0 to 4 and to refrain from changing the original structure where possible. Modification suggestions, such as changing the number of response options or splitting a category into several items, would be discussed in a subsequent process. In the second stage, the preliminary draft of the rating reference guide was forwarded to eight ICF experts for critical appraisal. The ICF experts reviewed the draft in terms of consistency 1) with the original definition and coding rules of ICF and 2) in the rating structure among the categories. The draft was then modified according to their comments. The rating reference guide was finalized after approval by the ICF experts. An English translation of the reference guide was developed by a multidisciplinary expert panel, following the procedure used to develop the English translation of the simple, intuitive descriptions.

### Reliability study

#### Participants

Once the rating reference guide was available, we examined its reliability. For this purpose, we recruited both patients who were receiving rehabilitation services at the university hospital and healthy individuals. The inclusion criteria were: 1) age of over 20 years old and 2) individuals or those whose family member could provide detailed information regarding the patient’s level of functioning.

Patients provided written, informed consent. In cases where a patient could not provide informed consent due to a cognitive disorder, their close relatives did so instead. Patients were excluded if they or their close relatives were unwilling to provide informed consent.

The study protocol was approved by the institution’s medical ethics committee.

#### Investigation of interrater agreement

In the reliability study, ratings were conducted by four independent specialists, a physiatrist, an occupational therapist and two physical therapists, whereby each patient was rated by two out of the four raters respectively. The raters received the developed rating reference guide to guide their rating. The ICF qualifier response options ‘8: not specified’ and ‘9: not applicable’ were maintained as rating options along with the ICF qualifier scale from 0 to 4. The sample size required for a rigorous reliability study was determined by the number of response options (five), the minimum value for the desired kappa coefficient (0.3 for every ICF category), the power (90.0%) and the alpha (0.05) we specified. These various elements dictated a minimum sample of 36 [17, 18]. Considering that there were several categories in the ICF Rehabilitation Set, such as d640 doing housework and d850 remunerative employment, do not apply to some of the inpatients, a certain number of missing data was anticipated. Accordingly, we set the sample size for each rater at 50 patients and obtained evaluation pairs for 100 patients.

#### Data analysis

Weighted kappa statistics were used to determine the interrater agreement between each pair of raters, and weighted kappa statistics with linear weights were calculated for each ICF category.

Since the response options 8 and 9 are not part of the ordinal scale ranging from 0 to 4, they were excluded from calculation of weighted kappa statistics. The analysis was conducted in each category of the activities and participation component of the ICF Generic-30 Set.

The standards for interpreting kappa statistics are as follows: ≦0.20, poor; 0.21–0.40, fair; 0.41–0.60, moderate; 0.61–0.80, substantial; and≧0.81, excellent.^22^ The response options ‘not specified’ and ‘not applicable’ were not included in the kappa statistics and were considered missing data.

## Results

### Development of simple, intuitive descriptions

The consensus conference was held in November 2016 in Nagoya, Japan. A total of 21 experts from all regions of Japan and different clinical areas of expertise were assembled to participate in a consensus conference. This group included physiatrists; physical, occupational, and speech therapists; and nurses. Two officers from the Ministry of Health, Labour and Welfare participated as observers. As a result of the predefined process, three initial proposals were accepted in the first step, and 20 proposals were accepted in the second step. The remaining seven ICF categories were decided in the final vote. All participants consented to the final, simple, intuitive descriptions. The English translations of the final versions are shown in Table 1.

**Table 1:** The Japanese version of the simple, intuitive descriptions of the ICF categories

Code	Title ENG	Simple intuitive descriptions
b130	Energy and drive functions	Mental functions that cause self-driven activities in daily life.
b134	Sleep functions	Necessary and sufficient sleep
b152	Emotional functions	Mental functions that control emotions appropriately
b280	Sensation of pain	Existence of pain
b455	Exercise tolerance functions	Physical capacity needed for activities of daily living
b620	Urination functions	Functions related to urinating stably in daily life
b640	Sexual functions	Mental and physical functions related to the sexual act
b710	Mobility of joint functions	Range and ease of movement of joints
b730	Muscle power functions	Muscle strength that is required for daily living
d230	Performing daily routines	Planning and carrying out daily activities
d240	Handling stress and other psychological demands	Coping with stress and/or distractions from tasks demanding responsibliity
d410	Changing basic body position	Changing body position such as standing up, sitting down, lying down, and squatting
d415	Maintaining a body position	Maintaining a body position such as sitting and standing
d420	Transferring oneself	Transferring onself, such as moving from a bed to a wheel chair
d450	Walking	Walking on level ground (including outdoors and rough roads)
d455	Moving around	Moving differently from walking such as going up and down the stairs, running, etc.
d465	Moving around using equipment	Moving around by using assistive devices such as wheelchairs, walkers, etc.
d470	Using transportation	Using various means of transportation to move around as a passenger
d510	Washing oneself	Cleaning, wiping and drying one’s whole body or body parts
d520	Caring for body parts	Caring for teeth, hair, beard, nails, skins, etc.
d530	Toileting	Managing urination, defecation, and menstruation appropriately in daily life, including cleaning oneself afterwards
d540	Dressing	Putting on and taking off clothes and footwear according to climatic and social conditions
d550	Eating	Eating safely by using necessary utensils
d570	Looking after one’s health	Performing self-management activities to ensure one’s own physical and mental well-being
d640	Doing housework	Doing housework (other than cooking) that is required in one’s daily life
d660	Assisting others	Assisting family members or others with their activities of daily living
d710	Basic interpersonal interactions	Interacting with people in an appropriate manner, such as showing respect, warmth, and consideration of different ideas and opinions
d770	Intimate relationships	Creating and maintaining close relationships between individuals, such as between husband and wife, or lovers etc.
d850	Remunerative employment	Engaging in remunerative work
d920	Recreation and leisure	Engaging in recreational or leisure activities

Four key topics emerged from a qualitative analysis of the discussion notes: [1] reconciling common clinical terms with the often detailed definitions of the original descriptions of ICF categories [2]; specifying the assumed level of functioning [3]; resolving several different aspects in a given category; and [4] handling the wording of the original definitions of ICF categories which are unfamiliar to Japanese clinicians.

#### Reconciling common clinical terms with the often detailed definitions of the original descriptions of ICF categories

Several participants indicated that numerous ICF definitions are excessively detailed for clinicians. For instance, the ICF category d450 Walking is described as ‘Moving along a surface on foot step by step so that one foot is always on the ground, such as when strolling, sauntering, walking forwards, walking backward or walking sideways’. However, from a clinical perspective, several raters in our study noted that this description is detailed but the usefulness of the description in clinical practice is constrained. For example, d450 has two subcategories, namely, d4502 Walking on different surfaces and d4503 Walking around obstacles, which refers to the walking outdoors or rough road. The description of these categories, however, are not reflected in the definition of d450. Walking on a flat floor indoors compared with the walking on a rough road outside might involve substantially different levels of difficulty, and any relevant rating for these activities should reflect the realities that clinicians deal with on a daily basis. As a result, the simple, intuitive description of d450 was agreed upon to be walking on level ground (including walking outdoors and walking on a rough road). This case also illustrates how examples were used in numerous descriptions to clarify their meanings and to define the scope of scoring in the corresponding categories.

#### Specifying the assumed level of functioning

Individuals can perform physical activities at a very wide array of levels. For example, for elite athletes, a slight decrease in muscle strength would be a critical problem. By contrast, such levels of difference might be almost irrelevant for patients after hip surgery. In light of this reality, our participants suggested that our new descriptions should leverage the common understanding of clinicians and facilitate the use of the ICF by including target levels of performance. For example, the simple, intuitive description of b730 Muscle power functions was refined to ‘Muscle strength that is required for daily living’. In this case, there was a discussion on whether the words ‘required for daily living’ should be included because this phrase is not included in the original definition of b730 Muscle power functions. However, considering that this description aims to help clinicians use the ICF and its rating system in daily clinical settings, this addition was readily justified by the participants.

#### Resolving several different aspects in a given category

Some participants indicated that ICF categories which included multiple elements in their descriptions should also be described in the simple, intuitive descriptions in detail, whereas others argued for keeping the descriptions simple and concise. The participants agreed eventually to include more detail when it was beneficial to enhance the clarity of the description of a given ICF category. For example, the category d410 Changing body positions was explained as ‘Changing body position such as standing up, sitting down, lying down and squatting’ because the scope of evaluation should be shown for clarification in this case.

#### Handling the wording in the original descriptions of ICF categories that is unfamiliar to Japanese clinicians

For many cultural and linguistic reasons, some ICF definitions are unclear to practitioners in Japan. For example, the ICF description of b130 Energy and drive functions is ‘General mental functions of physiological and psychological mechanisms that cause the individual to move towards satisfying specific needs and general goals in a persistent manner’. [2]. However, it was agreed that the phrase ‘to move towards satisfying specific needs’ is not intuitive for Japanese clinicians. Thus, in the Japanese version of simple, intuitive descriptions, this phrase was modified to ‘Mental functions that cause self-driven activities in daily life’ even though the phrase ‘self-driven activities in daily life’ is not part of the original ICF. However, it was used here because our participants considered that it nicely summarised subcategories such as b1301 Motivation, b1302 Appetite or b1303 Craving, all of which are more intuitive for Japanese clinicians.

### Development of the rating reference guide

In the next step, we developed the rating reference guide for the activity and participation categories in the ICF Generic-30 Set, based on the cognitive interviews with three rehabilitation experts (a physiatrist, a physical therapist and an occupational therapist) who rated nine patients with using simple, intuitive descriptions (Tables 2, 3). Three were acute patients, three subacute patients and three chronic patients. Five patients had a neurological disease, two had orthopaedic diseases and two had respiratory diseases. The cognitive interviews and the subsequent discussion suggested that the reference guide should reflect the different considerations for rating activity-related categories involving the execution of basic everyday tasks which individuals need to do for themselves to live, such as toileting and eating, versus rating participation-related categories involving engaging in activities related to a social context, such as interpersonal interactions and work. Consequently, the ICF categories were divided into two respective groups.

**Table 2:** Rating reference guide for activity-related categories Code

code	0: No problem	1: Mild problem	2: Moderate problem	3: Severe problem	4: Complete problem
d230					
Carrying out daily routine	May include: - Doing by him/herself without any problems	May include: - Doing by him/herself but being poor in planning activities - Doing by him/herself, but not active in planning activities	May include: - Doing partly with support for planning and doing daily activities	May include: - Doing largely with support for planning and doing daily activities	May include: - Doing completely with support - Being impossible to do
d240					
Handling stress and other psychological demands	May include: - Doing by him/herself without any problems	May include: - Doing by him/herself but requiring advice or encouragement from others to complete tasks	May include: - Doing partly with support and/or instruction from others	May include: - Doing largely with support and/or instruction from others	May include: - Doing completely with support - Being impossible to do
d410					
Changing basic body position	May include: - Doing by him/herself without any problems	May include: - Doing by him/herself with the use of orthosis, canes and/or handrail - Doing by him/herself with the supervision of others. - Doing by him/herself with a feeling of difficulty	May include: - Doing partly with support	May include: - Doing largely with support	May include: - Doing completely with support - Being impossible to do
d415					
Maintaining a body position	May include: - Doing by him/herself without any problems	May include: - Doing by him/herself with the use of orthosis, canes and/or handrail - Doing by him/herself with the supervision of others. - Doing by him/herself with a feeling of difficulty	May include: - Doing partly with support	May include: - Doing largely with support	May include: - Doing completely with support - Being impossible to do
d420					
Transferring oneself	May include: - Doing by him/herself without any problems	May include: - Doing by him/herself with the use of orthosis, canes and/or handrail - Doing by him/herself with the supervision of others. - Doing by him/herself with a feeling of difficulty	May include: - Doing partly with support	May include: - Doing largely with support	May include: - Doing completely with support - Being impossible to do
d450I					
Walking (indoors)	May include: - Doing by him/herself without any problems	May include - Doing by him/herself with the use of orthosis, canes and/or handrail - Doing by him/herself with the supervision of others. - Doing by him/herself with a feeling of difficulty	May include: - Doing partly with support	May include: - Doing largely with support	May include: - Doing completely with support - Being impossible to do
d450O					
Walking (outdoors and rough roads)	May include: - Doing by him/herself without any problems	May include - Doing by him/herself with the use of orthosis, canes and/or handrail - Doing by him/herself with the supervision of others. - Doing by him/herself with a feeling of difficulty	May include: - Doing partly with support	May include: - Doing largely with support	May include: - Doing completely with support - Being impossible to do
d455					
Moving around	May include: - Doing by him/herself without any problems	May include - Doing by him/herself with the use of orthosis, canes and/or handrail - Doing by him/herself with the supervision of others - Doing by him/herself with a feeling of difficulty	May include: - Doing partly with support	May include: - Doing largely with support	May include: - Doing completely with support - Being impossible to do
d465					
Moving around using equipment	May include: - Doing by him/herself without any problems	May include: - Doing by him/herself with the use of orthosis, canes and/or handrail - Doing by him/herself with the supervision of others - Doing by him/herself with a feeling of difficulty	May include: - Doing partly with support	May include: - Doing largely with support	May include: - Doing completely with support - Being impossible to do
d470					
Using transportation	May include: - Doing by him/herself without any problems	May include: - Doing by him/herself with the use of orthosis, canes and/or handrail - Doing by him/herself with the use of elevator - Doing by him/herself with the supervision of others. - Doing by him/herself with a feeling of difficulty	May include: - Doing partly with support	May include: - Doing largely with support	May include: - Doing completely with support - Being impossible to do
d510					
Washing oneself	May include: - Doing by him/herself without any problems	May include: - Doing by him/herself with the use of orthosis, canes and/or handrail - Doing by him/herself with the supervision of others. - Doing by him/herself with a feeling of difficulty	May include: - Doing partly with support	May include: - Doing largely with support	May include: - Doing completely with support - Being impossible to do
d520					
Caring for body parts	May include: - Doing by him/herself without any problems	May include: - Doing by him/herself with use of self-help devices - Doing by him/herself with the supervision of others - Doing by him/herself with a feeling of difficulty	May include: - Doing partly with support	May include: - Doing largely with support	May include: - Doing completely with support - Being impossible to do
d530					
Toileting	May include: - Doing by him/herself without any problems	May include: - Doing by him/herself with use of orthosis, self-help devices and/or handrail - Doing by him/herself with the supervision of others - Doing by him/herself with a feeling of difficulty	May include: - Doing partly with support	May include: - Doing largely with support	May include: - Doing completely with support - Being impossible to do
d540					
Dressing	May include: - Doing by him/herself without any problems	May include: - Doing by him/herself with use of orthosis and/or self-help devices - Doing by him/herself with the supervision of others - Doing by him/herself with limitations in wearable clothes - Doing by him/herself with a feeling of difficulty	May include: - Doing partly with support	May include: - Doing largely with support	May include: - Doing completely with support - Being impossible to do
d550					
Eating	May include: - Doing by him/herself without any problems	May include: - Doing by him/herself with use of orthosis and/or self-help devices - Doing by him/herself with the supervision of others - Doing by him/herself with limitations in food textures - Doing by him/herself with limitations in cutlery and/or tableware - Doing by him/herself with a feeling of difficulty	May include: - Doing partly with support	May include: - Doing largely with support	May include: - Doing completely with support - Being impossible to do
d570					
Looking after one’s health	May include: - Doing by him/herself without any problems	May include: - Doing by him/herself but requires advices or encouragement from others - Doing by him/herself with a feeling of difficulty	May include: - Doing partly with instructions from others	May include: - Doing largely with instructions from others	May include: - Doing completely with support - Being impossible to do

**Table 3:** Rating reference guide for participation-related categories

Code	0: No problem	1: Mild problem	2: Moderate problem	3:Severe problem	4: Complete problem
d640					
Doing housework	May include: - Doing housework by him/herself without any assisting device or support	May include: - Doing housework by him/herself with the use of orthosis, canes or handrails - Doing by him/herself with the supervision of others	May include: - Doing housework partly with support or being partly covered by support	May include: -Doing housework largely with support or being largely covered by support	May include: - Doing housework completely with support - Being impossible to do housework
d660					
Assisting others	May include: - Assisting others without restrictions or difficulties	May include: - Doing without restrictions, but with some difficulties	May include: - Doing partly with restrictions to what he/she can do to assist others	May include: - Doing largely with restrictions to what he/she can do to assist others	May include: - Being impossible to do anything to assist others
d710					
Basic interpersonal interactions	May include: - Interacting with people without apparent problems in showing respect, warmth and coordinating different opinions	May include: - Interacting with people without apparent problems in showing respect, warmth and coordinating different opinions but with some language difficulties - Interacting with people without apparent problems in showing respect, warmth and coordinating different opinions but the use of communicating devices	May include: - Interacting with people, but causes apparent problems in showing respect, warmth and coordinating different opinions at times	May include: - Interacting with people, but frequently causes apparent problems in showing respect, warmth and coordinating different opinions	May include: - Being impossible to interact with people
d770					
Intimate relationships	May include: - Creating and maintaining intimate relationships without problems	May include: - Having minimal problems which don’t fundamentally affect creating and maintaining the relationships	May include: - Rating between 1 and 3	May include: - Having serious problems that could apparently fundamentally affect creating and maintaining intimate relationships	May include: -Being impossible to create and maintain intimate relationship
d850					
Remunerative employment	May include: - Working without support or restrictions in terms of content, work time, and/or work intensity	May include: - Working without support or restrictions in content but with consideration in work time and/or work intensity. - Working without support or restrictions in content but with the use of assistive devices and/or in an assistive environment	May include: - Working partly with restrictions to work content - Working partly under support by others	May include: - Working largely with restrictions to work content - Working largely under support by others	May include: - Being impossible to work
d920					
Recreation and leisure	May include: -Doing leisure activities without restrictions or difficulties	May include: - Doing leisure activities without restrictions, but with some difficulties	May include: - Doing leisure activities partly with restrictions to the content	May include: - Doing leisure activities largely under restriction to the content	May include: - Being impossible to do leisure activities

#### *Ratings for activity-related ICF categories* (Table 2)

For activities such as toileting and eating, agreement was reached easily because links were drawn to existing clinical scales that address similar items, such as the Functional Independence Measure (FIM) [19] and the Barthel Index (BI) [20]. In activity-related ICF categories, the severity rating was largely determined by the requirements for human support, which basically reflects the style of existing clinical scales. However, this was broadened to include the need for many types of assistance devices and to also include the existence of mental barriers. In this section, there was substantial discussion regarding d450 Walking, because the functioning level required for walking indoors and walking outdoors and rough loads can be quite different [21, 22]. As a result, d450 Walking was split up into two items: walking indoors and walking outdoors and rough loads.

#### *Ratings related to participation-related ICF categories* (Table 3)

For the ICF categories referring to the participatory engagement of a person in daily life, it was agreed that a rating based solely on the degree of assistance required would not be appropriate. It is possible that some individuals who have difficulty in executing a task would be able to execute the task with modifications but without needing assistance. For example, a patient with fibromyalgia may be able to work without assistance from others but only with a modified number of hours, i.e. part-time instead of full-time. In this case, if the rating was solely based on the need for assistance and not also on the required modification, the rating reflect better functioning than in reality. Thus, it was agreed that the guide should consider both the restrictions in execution as well as the support required. The description of possible restriction was developed from the results of cognitive interviewing. The support required for the participation-related categories and for the activity-related categories were described similarly.

For d710 Basic interpersonal interactions and d770 Intimate relationships, the rating guide was developed differently. While some patients in some cases need support or have restrictionin in these types of interpersonal relationships, some do not. Thus, the rating guide for these categories were worded more broadly, and focused on the problem in the interaction itself rather than on the support the patient needs. The rating reference guide for d710 referred to “apparent problems in showing respect, warmth and coordinating different opinions”, and for d770 Intimate relationships refers to the problems that “apparently fundamentally affect creating and maintaining intimate relationships” .

Once the draft of the rating reference guide was available, the ICF experts raised further concerns regarding possible inconsistencies among the categories and with the original coding guideline for ICF. For example, there was some inconsistency in the wording within the activity-related categories even though the content was quite similar. Thus, the guide for those categories was modified to be as similar as possible in terms of wording. There were also several cases with the inconsistency with the original coding guideline or the simple, intuitive descriptions. In such cases, the reference guide was modified to avoid any discrepancy with the original ICF and simple, intuitive descriptions.

### Interrater reliability

Of the 100 patients recruited 84 were receiving rehabilitation services in the university hospital and 16 were healthy individuals over 65 years old. Sixty-five were males, and 35 were females; 55 patients had neurological diseases, 15 patients had orthopaedic diseases, 10 patients had cardiopulmonary diseases and four patients had various other issues (mostly renal and gastric in nature). The median days after onset was 58 (ranged 1 to 6403). The mean age of our subjects was 66 ± 17 years.

Missing values, including response options ‘not specified’ and ‘not applicable’, of more than 5% were present in 11 of the 21 ICF categories. No missing values were observed in seven categories.

Table 4 shows interrater reliability with percentages for complete agreement and kappa statistics (using linear weights) for individual ICF categories. The mean interrater agreement for the categories was 75.4% (ranging from 49.4 to 88.9%) indicating substantial agreement. Weighted kappa statistics showed a reliability of 0.6 or higher in all categories and 0.8 or higher (substantial agreement) in four of the categories (ranging from 0.61 to 0.85).

**Table 4:** Results of interrater reliability study

		Complete agreement	Weighted kappa (linear weight)	Missing values
d230	Carrying out daily routine	70.9%	0.61	21
d240	Handling stress and other psychological demands	74.4%	0.70	14
d410	Changing basic body position	81.0%	0.81	0
d415	Maintaining a body position	81.0%	0.79	0
d420	Transferring oneself	81.8%	0.79	1
d450 I	Walking (indoors)	80.6%	0.74	2
d450 O	Walking (outdoors and rough roads)	83.1%	0.82	17
d455	Moving around	73.5%	0.73	1
d465	Moving around using equipment	64.9%	0.72	29
d470	Using transportation	77.1%	0.67	41
d510	Washing oneself	70.0%	0.75	0
d520	Caring for body parts	74.0%	0.72	0
d530	Toileting	75.0%	0.78	0
d540	Dressing	75.0%	0.76	0
d550	Eating	88.9%	0.85	1
d570	Looking after one’s health	69.4%	0.63	15
d640	Doing housework	72.7%	0.73	43
d660	Assisting others	80.9%	0.84	46
d710	Basic interpersonal interactions	76.0%	0.66	0
d770	Intimate relationships	82.8%	0.68	30
d850	Remunerative employment	76.5%	0.77	37
d920	Recreation and leisure	49.4%	0.61	19

## Discussion

This paper described the development of several steps toward the development of a practical tool to foster the implementation of the ICF amongst clinicians in Japan. The first step in this process was to develop Japanese interpretations of simple, intuitive versions of the descriptions found in the ICF Generic-30 Set by using a method described in previous studies [8, 9]. Second, for each of the categories in this set, a ICF-based clinical data collection tool for rating the problems related to each category was developed by having both clinicians and researchers suggest ratings, discuss them in detail and eventually reach an agreement on a rating referencing guide. Finally, interrater reliability was tested for the resulting data collection tool. The results showed good to excellent reliability across different raters.

### Development of simple, intuitive descriptions

The Japanese version of simple, intuitive descriptions was largely developed via processes established in previous studies [8, 9]. However, the discussion in the consensus conference resulted in several differences from the previous versions. One difference was the omission of several words that were considered redundant for clinicians. For example, the description of d450 Walking in the Italian version is ‘Moving in an upright position step by step and always maintaining support on the ground,’ which is consistent with the original definition [9]. In contrast, the Japanese version is ‘Walking on level ground (including outdoors and rough roads)’, which omits the explanation regarding walking itself but adds information regarding subcategories.

There were also several differences compared with the previous versions that reflect the differences in language and culture. For example, in the Japanese version, new wording is used in the descriptions of several categories, where the direct translation of the original description of an ICF category into Japanese seemed to cause confusion for clinicians due to unfamiliar wording. These approaches taken in this study was to step beyond the mere simplification of the original descriptions. Nevertheless, efforts were made during the consensus conference to be consistent with the essential concept of the original descriptions.

### Development of the rating reference guide

Our effort to develop a clinically useful tool involved the development of a rating reference guide for the activity and participation categories contained in the ICF Generic-30 Set. In the development of this reference guide, participants distinguished between activity-related categories and participation-related categories.

Our guide regarding activity-related categories is mainly based on the need for human support. This approach is similar to other clinical scales, such as the FIM or BI, which are commonly used in rehabilitation clinics and have ratings that are mainly determined with regard to the degree of personal assistance required. This is possibly due to the fact that the guide was developed based on the cognitive interviewing of clinicians who are familiar with those clinical scales. Moreover, the patient’s dependence on the human support with regard to the activities of daily living may be gage of severity of a problem as dependence on human support strongly reduces the self-efficacy and quality of life (QoL) and also increases mental stress of patients [23–26]. Although an indirect association, reduced self-efficacy and QoL and mental stress can be seen as potential contributors to problems experienced in activity-related categories. Considering this, the amount of human support required along with other factors, such as the use of assistive devices and patient’s feelings of difficulty, informs the rating on activity-related categories.

On the other hand, the ratings related to participation were developed on the basis of both the limitations of what patients can do and the level of support they require. Many daily activities are not necessary for survival but add greatly to the perceived QOL of patients, such as participation in leisure activities [27]. Any restrictions on such actions, i.e. not just those evaluated with activity of daily living (ADL) scales such as the FIM, should also be considered in any patient-centred evaluation of functioning. Both the degree of dependency in daily activities and any constraints of participation should be considered limitations of self-determination [28].

### Interrater reliability study

Our interrater reliability testing of the ICF-based clinical data collection tool developed in this study showed that this instrument would be reliable for use in a clinical setting with a weighted kappa coefficient of over 0.60, thus demonstrating substantial agreement across different experts who used the ICF-based clinical data collection tool [29]. The good to excellent interrater reliability found in this study also supports the use of a rating reference guide. These findings are in line with a recent study that has shown that using the ICF qualifiers as a simple 0 to 4 rating scale is reliable [12]. In contrast, studies in which the ICF qualifier ratings were applied without guidance resulted in low reliability [10, 11].

We found that with our ICF-based clinical data collection tool, the kappa statistics in ADL-related categories, such as d450 Walking or d530 Toileting, were notably higher (> 0.70) than our weighted average kappa value. These better results may be driven by how we extensively incorporated actual clinical practice and descriptions of ICF categories with which practitioners are highly familiar into the design of our ICF-based clinical data collection tool .

On the contrary, we had several categories with relatively low kappa statistics, such as d230 Carrying out daily routine. This result is likely due to clinicians being less familiar with using rating scales for such concepts. Considering that many clinicians in Japan are unfamiliar with concept of some ICF categories, the need for an effective and simple reference system seems even more apparent.

Moreover, the setting in which the rating reference guide was developed was an inpatient rehabilitation setting. This frame of reference may make the rating of d230 Carrying out daily routine more abstract than walking or toileting. Nevertheless, the weighted kappa statistics for the ICF-based clinical data collection tool were still higher than 0.60, which is comparable to most standard clinical scales used in rehabilitation clinics [30, 31].

Compared with the low reliability shown in previous studies, our results will better enable the robust clinical use of the ICF as a clinical data collection tool. Our weighted kappa statistics are comparable to scales such as the FIM, which has a detailed explanation for each response option related to an item [32, 33]. Overall, the metrics measuring the effectiveness of our ICF-based clinical data collection tool would support the clinical use of our rating instrument in daily clinical practice.

### Practical implications

This study aimed to develop a simple and reliable ICF-based clinical data collection tool consistent with the original ICF coding guidelines and based on the reasoning of rehabilitation professionals. This effort included the development of intuitive descriptions and a rating reference guide for which the reliability of the categories regarding patient activity and participation in daily living was confirmed. This clinical data collection tool has several strengths: First, it goes beyond the traditional description of ADL and builds upon the ICF as a frame of reference. It is comprehensive in that it includes ICF categories from 7 out of the 9 chapters of the activities and participation component. Thus, this tool may help to capture the problems in patients’ functioning more comprehensively, and with more options for describing patients’ functioning, possibly also more individualized. Second, the development of simple, intuitive descriptions was based on the consensus of rehabilitation professionals with various backgrounds. This would facilitate the use of this tool as a bridge between various rehabilitation professionals. It can be used not only to evaluate the extent of the problems or to detect the changes in patient status, but also for sharing information on the patients’ functioning. Furthermore, guiding clinicians of various disciplines to rate using the same reference guide would encourage them to view patients in a mutual way. As a whole, this can positively influence the real-life implementation of this ICF-based clinical tool in clinics. Lastly, the rating reference guide resulted from the systematic analysis of the reasoning rehabilitation professionals apply when rating actual patients. The high interrater reliability of our ICF-based clinical data collection tool supports this comprehensive development process and is promising with regards to the wider use of the ICF in clinics.

### Limitations

Our reference guide was developed by having three clinicians evaluate nine patients by using the ICF Rehabilitation Set. This design involves a small dataset. However, our method involves analysing the actual thinking process of clinicians when they are evaluating patients whose conditions range from chronic to highly acute. Our interviewer asked the three clinicians the reason for their selection of a particular response for each category, and the final outcome was based on the 27 questions and answers in each category. To help keep our investigation tightly focused, we decided to limit the amount of data we collected and evaluated. Despite the several layers of review of the rating reference guide to confirm its robustness, further investigation into its applicability in a diverse rehabilitation settings is warranted.

## Conclusions

In this project, we developed a user-friendly and reliable ICF-based clinical data collection tool for Japan that can be implemented in various clinical settings. This tool comprises intuitive descriptions are consistent with the ICF, and are easy to understand for clinicians. and the use of these clinician-friendly descriptions and the companion response items within a robust scoring system are further supported by a rating reference guide developed in this study. A further examination of the utility and limitations of the ICF-based clinical data collection tool developed in this study is warranted, including its applicability in clinical practice all of over the world.

This user-friendly and reliable ICF-based clinical data collection tool was developed specifically for Japan. It also represents a tailored version of ClinFIT – Clinical Functioning Information Tool, the International Society of Physical and Rehabilitation Medicine’s universal and non-proprietary ICF-based tool for clinical assessment and reporting of patient functioning in rehabilitation. ClinFIT can be tailored for a specific purpose, context (e.g. country) or patient population (e.g. based on age or health condition). Indeed, other countries may find this tailored version of ClinFIT suitable for their context.

## Data Availability

The datasets used and/or analysed during the current study are available from the corresponding author on reasonable request.

## Abbreviations

ADL: activities of daily living
BI: Barthel Index
FIM: Functional Independence Measure
ICF: International Classification of Functioning, Disability and Health
QOL: Quality of Life
